# Prevalence and molecular characterisation of *Balantioides coli* in pigs raised in Italy

**DOI:** 10.1007/s00436-025-08452-w

**Published:** 2025-01-16

**Authors:** Carolina Allievi, Francisco Ponce-Gordo, Luca Villa, Alessandro Zanon, Marco Valleri, Sergio Aurelio Zanzani, Michele Mortarino, Maria Teresa Manfredi

**Affiliations:** 1 Department of Veterinary Medicine and Animal Sciences, Università Degli Studi Di Milano, Via Dell’Università, 6, 26900 Lodi, Italy; 2 Research Laboratory of Animal Parasitic Diseases and Zoonoses (ParVetLab), Università Degli Studi Di Milano, Via Dell’Università 6, 26900 Lodi, Italy; 3 Department of Parasitology, Faculty of Pharmacy, Complutense University of Madrid, Plaza Ramón y Cajal S/N, 28040 Madrid, Spain

**Keywords:** *Balantioides coli*, Zoonosis, Pigs, Copromicroscopic techniques, Genetic polymorphism

## Abstract

*Balantioides coli* is the only ciliated protist of both human and veterinary interest and colonises the large intestine of several hosts, including humans and pigs. Given the scarcity of data on *B. coli* circulation in pigs in Italy, a study was planned to record its prevalence and genetic types and compare the analytical sensitivity of two copromicroscopic techniques. For this purpose, 880 faecal samples were collected from pigs raised in 22 farms located in northern Italy and analysed with the sedimentation and the FLOTAC® dual technique, employing sodium chloride and zinc sulphate flotation solutions, and the Cohen’s kappa coefficient was calculated to assess the agreement between these methods. Conventional PCR to amplify the ribosomal internal transcribed spacer region (ITS) was subsequently performed on 22 samples, one for each farm, which were positive for *B. coli* cysts by sedimentation; when more than one sequence was detected in the chromatograms, the PCR products were cloned. Overall, 813 samples were positive for *B. coli* cysts by sedimentation (92.4%), and moderate concordance with the zinc-based FLOTAC® technique was observed, whereas the agreement was slight with the salt-based FLOTAC® technique. Among the 22 sequenced samples, type B was amplified in 19 samples, type A in two samples, and both A and B types were co-expressed in one sample. This study demonstrated a high prevalence of *B. coli* in Italian pigs; further insights are needed to trace its distribution and genetic polymorphism in other pig production categories and in humans, defining its public health significance.

**Supplementary Information:**

The online version contains supplementary material available at 10.1007/s00436-025-08452-w.

## Introduction

*Balantioides coli* (Phylum Ciliophora) is a ciliated protist that infects several hosts, including pig, non-human primates, birds, rodents and humans, posing an important risk of zoonotic transmission. It colonises the large intestine and is transmitted via the faecal‒oral route, through contaminated food or water or direct contact with infected animals (Schuster and Ramirez‒Alvila 2008; Ahmed et al. 2019; Ponce‒Gordo and García‒Rodríguez 2021). The cyst, which represents the only infective stage, can survive in the environment for a few days at room temperature and under favourable conditions, such as in pig faeces, for weeks; in contrast, the trophozoite stage dies a few hours after faecal expulsion (Schuster and Visvesvara 2004; Pomajbíková et al. 2010; García-Rodríguez et al. 2022).

In humans, balantidiasis has been reported in many countries, mainly in Asia, South America and Africa, but also in Europe, especially in individuals in close contact with pigs and with a compromised immune system (Ferry et al. 2004; Maino et al. 2010; da Silva et al. 2021; Ponce-Gordo and García-Rodríguez 2021). Clinical symptoms are characterised by dysentery, but cases of extraintestinal infection involving the peritoneum, urogenital tract and lungs may also occur (Sharma and Harding 2003; Kapur et al. 2016; Kaur and Gupta 2016; Almaw et al. 2022). In developed countries, the consumption of raw or undercooked meat could increase the risk of human infection, as *B. coli* inhabits the last intestinal tract of animals and meat contamination could occur during the evisceration phase in slaughterhouses due to poor hygiene practices, which could also lead to cross-contamination between carcasses. On the other hand, in developing countries, *B. coli* is considered a waterborne parasite through contamination of recreational or drinking water because of failures in water supply and dispersal of faeces (Giarratana et al. 2021).

In pigs, this protist generally does not cause clinical symptoms, but it can act as an opportunistic pathogen of secondary irruption, inducing mucosal ulceration, dysentery and growth retardation, and causing significant economic losses for the intensive pig industry (Ahmed et al. 2019; Giarratana et al. 2021; Szczotka-Bochniarz et al. 2021).

Currently, there is no standardised detection method for *B. coli*; as the size and morphological characteristics are distinctive, tests based on direct observation of faecal smears, flotation and sedimentation techniques, are commonly used. To date, the copromicroscopic method with the highest analytical sensitivity is sedimentation, especially compared with flotation, where the use of different solutions could cause deformation of *B. coli* cysts, preventing their microscopic identification (da Silva Barbosa et al. 2016; Ponce-Gordo and García-Rodríguez 2021). Furthermore, recent advances in molecular techniques have enabled the study of several features of *B. coli*, particularly two fragments of ribosomal RNA (rRNA) genes, including the small subunit rRNA (SSU rRNA) and the ITS1-5.8S rRNA-ITS2 (ITS) region, have been developed as molecular markers. The SSU rRNA gene was used to identify *B. coli* at the species level, while the ITS region allowed the differentiation of two main genetic types, namely, A and B (Ponce-Gordo et al. 2008, 2011). Each major type, in turn, comprises some variants, depending on their sequence similarity, designated A0, A1 and A2 for type A and B0 and B1 for type B. To date, types A and B and sequence variants A0, A1, B0 and B1 have been reported in pigs; in humans, only type A with sequence variants A0 and A2 (Ponce-Gordo et al. 2011).

Considering the zoonotic potential of *B. coli*, the limited data available on its circulation in pig farms and the fact that, to the best of the authors’ knowledge, its molecular characterisation has never been carried out in Italian pigs, the aims of this study were to determine the prevalence of this parasite, compare the analytical sensitivity of two different copromicroscopic methods, and trace the ITS genetic types using molecular techniques.

## Materials and methods

### Sampling and data collection

Pig sampling was performed as previously described for an epidemiological survey on gastrointestinal parasites in pigs raised in intensive systems (Allievi et al. 2024). Briefly, a minimum sample size of 385 faecal samples was calculated using a statistical tool (Epidemiological Calculators, www.epitools.ausvet.com.au), considering a population of fattening pigs in northern Italy of about 2 million animals, a 50% expected prevalence, a 95% confidence level and a 5% desired absolute precision (National Zootechnical Database, https://www.vetinfo.it/). The sampling was carried out from April to October 2023, and a total of 880 faecal samples, each weighing approximately 20 g, were collected from 22 fattening pig farms located in northern Italy, in an area between the regions of Lombardy (18 farms), Piedmont (3 farms) and Emilia-Romagna (1 farm). All farms were intensive and pigs, commercial hybrids of Large White and Landrace breeds, were raised indoors and grouped into several pens (each pen with 20 animals). In each farm, 20 individual faecal samples were taken from the rectal ampulla of apparently healthy pigs from different pens in two collection sessions. The first sampling (T1) was performed at the entry of the fattening cycle, when the pigs were three months old and weighed 35 kg on average, whereas the second sampling (T2) was carried out about one week before the slaughter date, when the animals were nine months old and weighed 170 kg on average. Thus, 440 faecal samples collected at T1 and 440 collected at T2 were placed in individual sterile plastic containers and transferred to the laboratory at refrigeration temperature (+ 4 °C). Copromicroscopic analysis of each sample was performed in the two days immediately following collection, while one aliquot of each faecal sample was prepared and stored at − 20 °C for subsequent molecular analysis.

At the sampling time, data regarding farm management were collected by interviewing the farmer: the application of an all-in/all-out system (yes/no), which refers to the sanitary vacuum of the farm before a new fattening cycle, the type of floor (full, mixed, slatted), and the type of watering (aqueduct water, well water). In addition, an animal cleanliness score was defined on the basis of the faecal soiling of the bodies, according to the scheme proposed by Classyfarm, a risk categorisation system funded by the Ministry of Health and implemented by the Istituto Zooprofilattico Sperimentale of Lombardy and Emilia-Romagna (https://www.classyfarm.it/): 0, the soiled body surface does not exceed 20%; 1, the soiled body surface is between 20 and 50%; 2, the soiled body surface exceeds 50%.

### Copromicroscopic and molecular analysis

Firstly, the sedimentation was performed on all faecal samples, following the procedure described by Foreyt (2001); the sediments were observed unstained in a Petri dish under a stereomicroscope at 50 × magnification. On the 440 samples collected at T2, flotation was also performed using the FLOTAC® dual technique: two flotation solutions, FS2 (sodium chloride, NaCl; specific gravity = 1.200) and FS7 (zinc sulphate, ZnSO_4_; specific gravity = 1.350), which are valid for the detection of helminths (eggs or larvae of nematodes, eggs of cestodes and trematodes) and intestinal protozoa (cysts and oocysts), were employed (Cringoli et al. 2010).

DNA extraction was performed on 22 faecal samples that were positive for the presence of *B. coli* cysts (one for each farm) employing the sedimentation technique. Genomic DNA was extracted from 200 mg of faeces using the QIAamp® Fast DNA Stool Mini Kit (QIAGEN, Hilden, Germany), following the manufacturer’s instructions. DNA samples were subsequently subjected to conventional PCR, and the 3′ end (~ 117 bp) of the SSU-rRNA gene, the ITS region and the start of the 5′ end (~ 28 bp) of the LSU-rRNA gene of *B. coli* were amplified using the forward primer B5D and the reverse primer B5RC (Suppl. File 1) (Ponce-Gordo et al. 2011). PCRs were performed using illustra™ PuReTaq Ready-To-Go PCR Beads (Merck KGaA, Darmstadt, Germany; now Cytiva Lifescience™ illustra™ PuReTaq Ready-To-Go™, Fisher Scientific, Pittsburg, USA) in a total volume of 25 µl, including 5 µl of the template DNA solution and 1.5 µl of each 5 mM primer solution. A Mastercycler Gradient 5331 thermocycler was used to control the cycling conditions (Suppl. File 1). PCR amplicons were visualized in 1% low-melting agarose gels stained with Pronasafe (Condalab, Torrejón de Ardoz, Spain), and those showing bands of the expected size, approximately 500 bp, were purified with the NucleoSpin® Gel and PCR Clean-up Kit (Macherey–Nagel, Düren, Germany). Then, the purified PCR products were sent for sequencing with the B5D primer to a commercial service (Microsynth Seqlab, Göttingen, Germany). The obtained chromatograms were processed with ChromasPro software, version 2.1.10 (Technelysium Pty. Ltd. South Brisbane, Australia), and the sequences were compared with those available in the GenBank database using the BLASTn algorithm. When more than one sequence was detected in the chromatograms, the PCR products were cloned using the CloneJET™ PCR Cloning Kit (Thermo Fisher Scientific, Waltham, USA). The plasmid DNA was extracted from the positive colonies using the GeneJET™ PCR Purification Kit (Thermo Fisher Scientific, Waltham, USA) and visualized in 1% low-melting agarose gels stained with Pronasafe. At least 5 clones per cell were sent for sequencing to the Microsynth Seqlab using the B5D primer; the obtained chromatograms were subsequently processed with ChromasPro software, and the sequences were compared with those available in the GenBank database via the BLASTn algorithm.

All the sequences were aligned using the ClustalW algorithm as implemented in ClustalX 2.1 (Larkin et al. 2007); the *B. coli* sequences JF444762, JF444763m JF444759, JF444764 and JF444765, corresponding to the sequence variants A0, A1, A2, B0 and B1, respectively, were included for reference purposes; and the sequence of *Buxtonella sulcata* JQ073387 was used as outgroup. The 5′ and 3′ ends (corresponding to the 3′ end of the SSU-rRNA gene and the 5′ end of the large subunit rRNA gene, respectively) were trimmed to keep only the ITS sequences; the final alignment included 29 sequences and 371 positions. Phylogenetic analysis was carried out in MEGA-X (Kumar et al. 2018). The relationships between sequences were inferred via the Maximum Likelihood (ML) method and the Tamura‒Nei model of nucleotide substitutions (Tamura and Nei 1993), which was selected as the best model based on the Akaike Information Criterion (AIC) and the Bayesian Information Criterion (BIC), as implemented in MEGA-X.

### Statistical analysis

*Balantioides coli* infection was evaluated by qualitative analysis (presence/absence), and an animal was considered infected if at least one *B. coli* cyst was detected performing the sedimentation technique. The parasite prevalence in each farm was calculated according to Bush et al. (1997), and farm management data (application of an all-in/all-out system, type of floor and type of watering) and animal cleanliness score were assessed as risk factors for *B. coli* occurrence and introduced into generalized linear models (GLMs) as categorical independent variables, while parasite status (positive/negative) was considered as the dependent variable. Moreover, for the faecal samples analysed at T2, the Cohen’s kappa coefficient was calculated to evaluate the agreement between the copromicroscopic methods, i.e. between the sedimentation and the FLOTAC® dual technique, considering both flotation solutions, and the significance level was set at 5%. The kappa coefficient was interpreted according to Landis and Koch (1977): < 0, poor agreement; 0–0.20, slight agreement; 0.21–0.40, fair agreement; 0.41–0.60, moderate agreement; 0.61–0.80, substantial agreement; and 0.81–1.0, near perfect agreement.

Statistical analysis was performed using SPSS version 28.0.1.1 (IBM, Chicago, IL, USA).

## Results

**Fig. 1:**
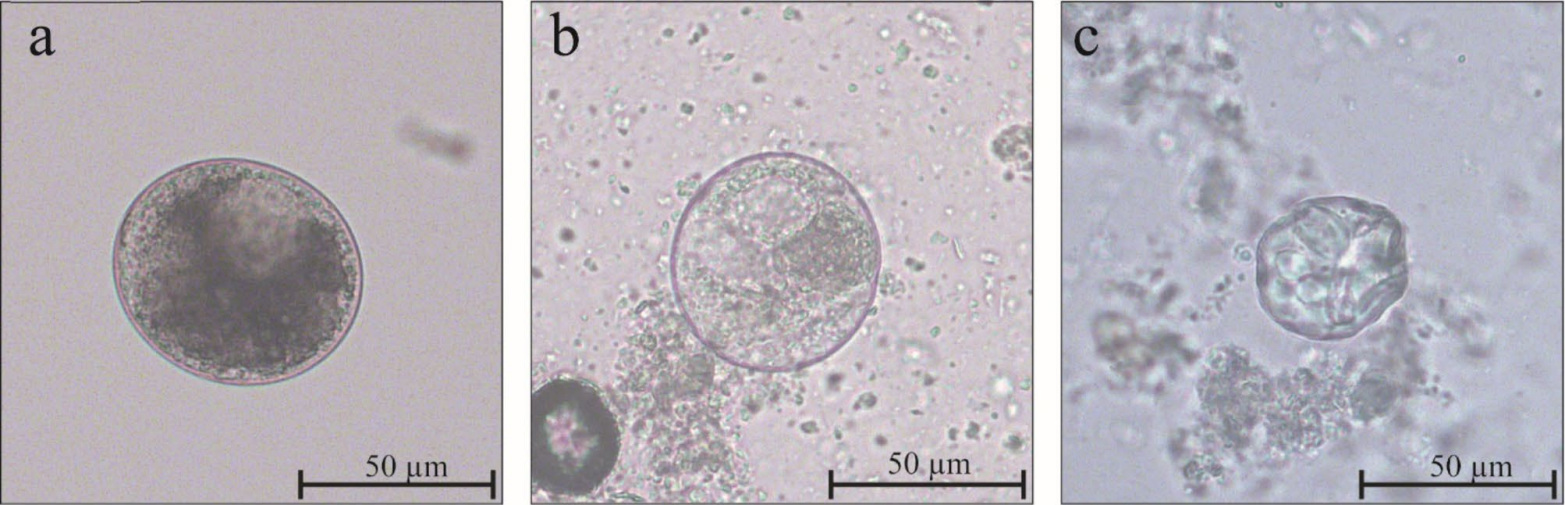
*Balantioides coli* cysts isolated from faecal samples using different copromicroscopic techniques. **a** Cyst isolated by sedimentation technique. **b** Cyst isolated by FLOTAC® with NaCl solution. **c**: Cyst isolated by FLOTAC® with ZnSO_4_ solution. All images are at 200 × microscope magnification

813 out of 880 samples were positive for the presence of *B. coli* cysts using the sedimentation technique, with an overall prevalence of 92.4% (Confidence interval (CI): 90.4–94%). In the first sampling session, 391 out of 440 samples were positive (88.9%, CI: 85.5–91.6%), whereas in the second sampling session the number of positive animals increased to 422, with a prevalence of 95.9% (CI: 93.6–97.6%). The 440 faecal samples collected in the second sampling session were also analysed by the FLOTAC® dual technique: 377 positive samples out of 440 were identified (85.7%, CI: 82.1–88.8%) with FS7 (ZnSO_4_) solution, while positives dropped to 39 (8.9%, CI:6.4–11.9%) with FS2 (NaCl) solution (Table 1). The morphology of *B. coli* cysts remained intact when the sedimentation and the FLOTAC® technique with sodium chloride solution were used; while some deformation was observed in the cysts obtained with the FLOTAC® technique employing zinc sulphate solution (Fig. 1).

**Table 1:** Successful detection of *Balantioides coli* cysts in pigs raised in Italy using different copromicroscopic techniques and concordance of results by Cohen’s kappa (*k*) coefficient (*n* = 440)

Technique	No. of positive samples/total	Prevalence % (95% CI^a^)	k coefficient	*p* value
Sedimentation	422/440	95.9% (93.6–97.6)	0.407	0.000^*^
FLOTAC® with ZnSO_4_ solution	377/440	85.7% (82.1–88.8)		
Sedimentation	422/440	95.9% (93.6–97.6)	0.008	0.0883
FLOTAC® with NaCl solution	39/440	8.9% (6.4–11.9)		

^a^*CI*, confidence interval

^*^Value statistically significant (*p* value < 0.05)

The Cohen’s kappa coefficient was statistically significant in the comparison between sedimentation and the FLOTAC® technique with the employment of zinc sulphate flotation solution, showing moderate statistically significant agreement (0.47, *p* value < 0.05). In contrast, the concordance was slight between sedimentation and the FLOTAC® technique with sodium chloride flotation solution (0.15), and the kappa coefficient was not statistically significant (*p* value > 0.05) (Table 1).

Most of the sampled farms applied all-in/all-out systems (16/22) and had full flooring (12/22) and drinking systems based on well water (20/22); the cleanliness score was mainly between 1 and 2, indicating high faecal contamination of the animals’ bodies (Suppl. Table 1). *Balantioides coli* was identified in all examined farms in both sampling sessions: at T1, five farms were 100% positive (20 out of 20 positive samples), and the lowest number of positive samples recorded at the farm level was 12 out of 20. At T2, 13 farms were 100% positive, and the lowest number of positive samples found at the farm level was 16 out of 20. For almost all farms, an increase in prevalence was observed during the second investigation, i.e. at the end of the fattening cycle, except for farms 2, 8, 10, 12 and 17, where positivity values were slightly higher in the first session, and farms 1 and 18, which had the same number of positive samples in both sampling times (Suppl. File 2). In addition, a greater increase in positivity was observed from T1 to T2 in farms with a full floor, which did not apply the all-in/all-out system, administered well water, and with animal cleanliness scores between 1 and 2 (Suppl. Table 1). In the statistical analysis, none of the variables, including both farm management data and animal cleanliness score, was significantly associated with *B. coli* infection.

Among the 22 sequenced samples, ITS genetic type A was identified in two samples, and type B in 19 samples, while both types were co-expressed in one sample. The type A sequences corresponded to the variant A0 and had two variable sites (positions 94 and 277, Suppl. File 3), while the type B sequences were B0 and B1 and had seven variable sites (positions 10, 44, 89, 272, 273, 277 and 306, Suppl. File 3). Both samples expressing type A were from farms located in Lombardy (Table 2). In ten of the type B sequences, a mix of B0 and B1 variants was most likely present, as clear double peaks (indicated by ambiguity codes in Suppl. File 3) were observed in differential positions in the ITS1 fragment. For example, M(pos.10)/Y(45) can correspond to type B0: C(10)/C(45) and type B1: A(10)/T(45). In the sample where types A and B were co-expressed, variants A0 and B1 were identified in the cloned products (Table 2).

**Table 2:** Molecular genotyping of one positive sample for *Balantioides coli* for each examined pig farm in Italy

Farm code	Farm region	ITS sequence type	Sequence variant
1	Lombardy	B	B0
2	Piedmont	B	B0
3	Piedmont	B	B0
4	Lombardy	B	B0, B1
5	Lombardy	B	B0, B1
6	Piedmont	B	B0, B1
7	Lombardy	B	B0, B1
8	Lombardy	B	B0, B1
9	Lombardy	B	B0, B1
10	Lombardy	B	B0, B1
11	Lombardy	B	B0, B1
12	Lombardy	A	A0
13	Lombardy	B	B0
14	Lombardy	B	B0
15	Lombardy	B	B0
16	Lombardy	B	B0
17	Emilia-Romagna	B	B0
18	Lombardy	B	B0, B1
19	Lombardy	A, B	A0, B1
20	Lombardy	B	B0
21	Lombardy	A	A0
22	Lombardy	B	B0, B1

**Fig. 2:**
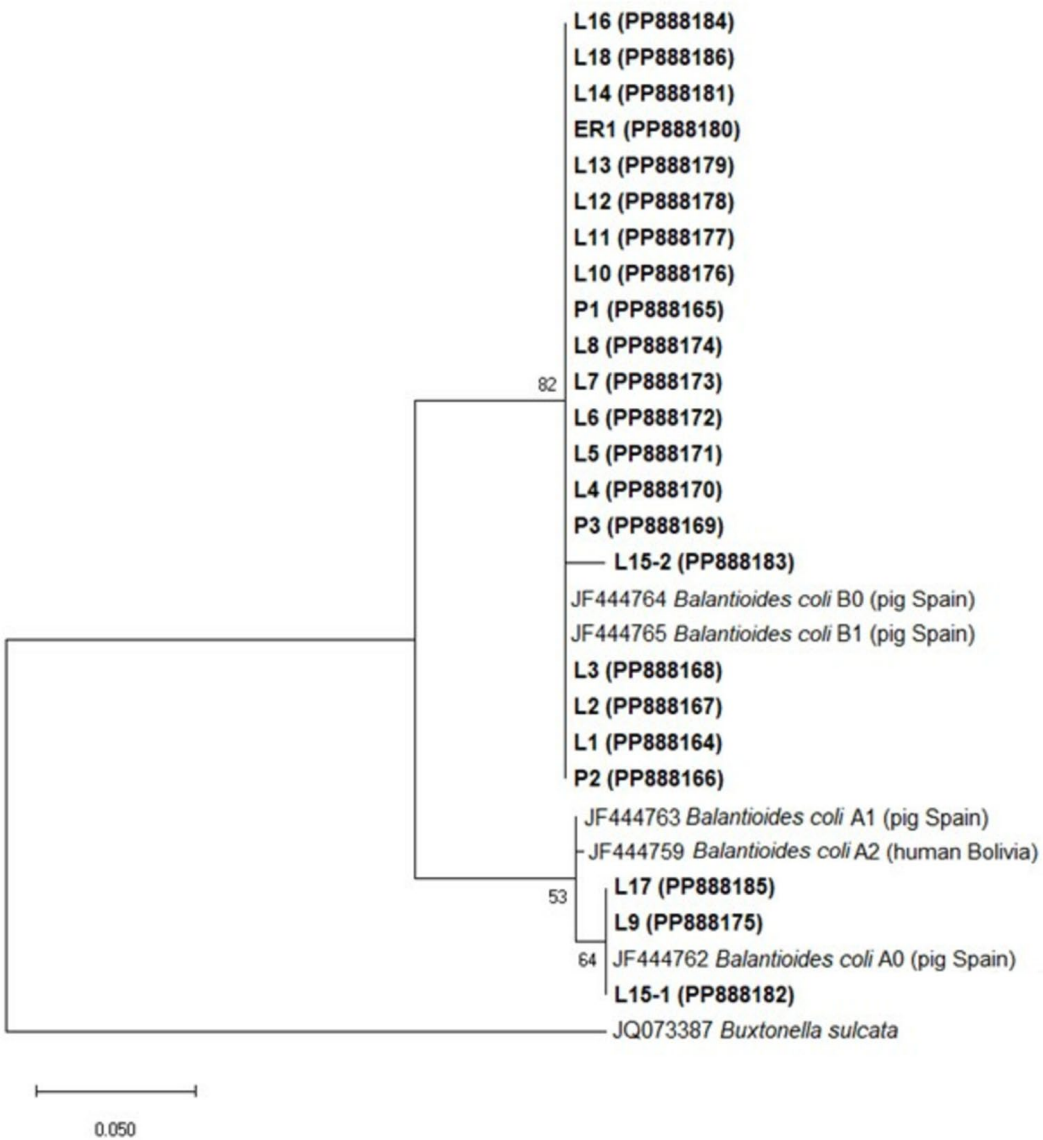
Phylogenetic relationships between the ITS sequences of 22 pig isolates of *Balantioides coli* inferred by the Maximum Likelihood method and the Tamura‒Nei model of nucleotide substitution; a discrete Gamma distribution (+ G = 0.1810) was used to model differences in evolutionary rate among sites. The number in the branches indicates the bootstrap support based on 1000 replicates. The sequence of *Buxtonella sulcata* JQ073387 was used as outgroup. The scale represents the number of substitutions per site. The sequences obtained in this study are marked in bold. The codes indicate the farms from each region: ER, Emilia-Romagna; L, Lombardy; P, Piedmont

The phylogenetic analysis clearly separated the sequences into two groups, one corresponding to type A and the other to type B (Fig. 2).

All the nucleotide sequences generated in the present study were submitted to GenBank under the following accession numbers: PP888164–PP888186.

## Discussion

The present study investigated the circulation and prevalence of *B. coli* in 22 fattening pig farms located in northern Italy, comparing two different copromicroscopic methods and tracing the molecular diversity of this parasite. The reported prevalence, 92.4%, was rather high, probably due to the intensive system of the selected farms, where the close contact between animals inside the pens and the reduced outdoor access could enhance its spread (Giarratana et al. 2021). Moreover, the positive values were slightly higher in the second sampling session, as prevalence of *B. coli* could increase with the increasing age of the animals (Hindsbo et al. 2000; Schubnell et al. 2016; Symeonidou et al. 2020).

Prevalences in fatteners raised in intensive systems, using copromicroscopic techniques, range from 47.2% to 60.3%, depending on the diagnostic method and flotation solution used (Weng et al. 2005; Schubnell et al. 2016; Sangioni et al. 2017; Symeonidou et al. 2020). In Italy, the only two recent studies were conducted in Sicily (southern Italy) and reported high values, although lower than those reported in our study, probably due to the low specific gravity of the flotation solution employed (sodium chloride, NaCl) (Giarratana et al. 2012, 2021).

According to the data obtained, sedimentation detected a higher number of samples positive for the presence of *B. coli* cysts. A moderate significant concordance (*p* value < 0.05) of the positive results was demonstrated with the FLOTAC® technique using the zinc sulphate solution; however, the deformation of the cyst wall due to the osmotic pressure exerted by the solution could negatively affect the analytical sensitivity of this technique. In this regard, a study conducted in Brazil demonstrated the poor performance in detecting *B. coli* cysts when using high specific gravity solutions. Specifically, out of 790 pig faecal samples analysed by both sedimentation and flotation with zinc sulphate, the former detected 18.4% of positive samples and the latter only 5.1%, because of morphological changes in the cysts that prevented their recognition (da Silva Barbosa et al. 2016). In contrast, employing sedimentation and the FLOTAC® technique with the sodium chloride solution, the cyst wall was intact; nevertheless, a slight concordance (*p* value > 0.05) between the methods was observed and significantly fewer positive animals were detected by FLOTAC® with the sodium chloride, confirming the low sensitivity of this solution for the detection of *B. coli* (Cringoli et al. 2010). Therefore, spontaneous sedimentation seems to be more sensitive, although this method has also limitations: it is operator dependent and the sediment is characterised by a heterogeneous distribution of parasitic elements; thus, a negative result could be related to the lack of cysts in the observed sediment but not necessarily to the negativity of the examined sample (Ahmed et al. 2019; Gonçalves et al. 2014; Pinilla et al. 2021; Pomajbíková et al. 2010; Ponce-Gordo and García-Rodríguez 2021).

Molecular identification targeting the rRNA gene could be used for the clinical diagnosis of *B. coli*, overcoming possible false positives/false negatives, and for phylogenetic analyses (Ponce-Gordo et al. 2008, 2011; Esteban-Sánchez et al. 2023). In our study, most of the samples showed ITS type B, as documented by other surveys, (Dashti et al. 2021; Li et al. 2020; Zhang et al. 2023) and, to a lesser extent, type A. Notably, each *B. coli* cell can simultaneously express multiple ITS genetic variants at the ribosomal level; so, it is not possible to use this variability to identify subpopulations, study host specificity and zoonotic transmissibility, or trace the epidemiology of the infection (Pomajbíková et al. 2013; Stensvold et al. 2021). To date, type A with the sequence variants A0 and A2 has been isolated in humans, but the expression of other ITS types cannot be ruled out, as only a single human isolate has been genetically studied and more samples of human origin should be analysed (Ponce-Gordo et al. 2011; da Silva Barbosa et al. 2017).

*Balantioides coli* circulates worldwide, especially in tropical and subtropical areas where high temperatures and poor environmental and sanitary conditions can increase its survival and spread (Yin et al. 2015; Ahmed et al. 2019). Nevertheless, there is a lack of updated data on its actual presence and distribution in both fresh produce, such as vegetables and fruit, and raw or undercooked meat (FAO and WHO 2014; Nalbone et al. 2021; García-Rodríguez et al. 2022). In this context, *B. coli* is included among the foodborne parasites that should be included on a priority list for the development of standardised control guidelines (Bouwknegt et al. 2018). The increase in pig herds may pose an emerging public health risk due to manure spreading on fields and watersheds, considering that water-associated outbreaks of balantidiasis have been reported in the literature (Karanis et al. 2007). In fact, contamination and subsequent consumption of untreated water, and unsafe handling of pig manure can lead to the acquisition of infections by common parasites in clinically healthy pigs, mainly involving personnel of the food chain (Solaymani-Mohammadi and Petri 2006; Karanis et al. 2007). In industrialised countries, *B. coli* plays a marginal zoonotic role, as the indoor pig housing and the use of manure containment systems have further reduced the risk of watershed contamination; however, even in such civilised settings, cases of infection in humans occur. For example, two clinical episodes have been reported in France, one in a pork butcher who presented with severe peritonitis; the other in an individual with dysentery. In both cases, faecal examination revealed the presence of numerous *B. coli* trophozoites and cysts. In Italy, the only reported clinical case concerned a man undergoing chemotherapy hospitalized for acute kidney injury after drinking contaminated water, and the examination of urinary sediment confirmed the presence of many *B. coli* trophozoites (Ferry et al. 2004; Maino et al. 2010; Bellanger et al. 2013). Thus, it is essential to implement appropriate measures to prevent balantidiasis, such as using water from protected sources, washing hands after handling pigs, and properly disposing of pig manure. Since *B. coli* cysts can survive for weeks in moist matrices, as faeces, direct use of manure as fertiliser should be limited, favouring a longer period of maturation or processing for biogas production (Schuster and Visvesvara 2004; Hampton et al. 2006; da Silva et al. 2021).

When evaluating the management characteristics of the farms included in our study (Suppl. Table 1), most of them administered well water to the animals, which is less sanitised and less controlled than aqueduct water, and could be more easily contaminated with *B. coli* cysts (Hampton et al. 2006; Sangioni et al. 2017). Numerous farms applied the all-in/all-out system, which is supposed to limit parasite circulation through a sanitary vacuum at the end of each fattening cycle. However, prevalence was high, highlighting the importance of combining the all-in/all-out procedure with proper cleaning and disinfection measures; such as the daily removal of faeces from pens, especially in farms with full flooring, as this type of flooring does not allow natural drainage of faeces and could increase environmental contamination (Pettersson et al. 2021).

The evaluation of the cleanliness score showed that most farms had animals with a rather high degree of faecal soiling, which could enhance the circulation of *B. coli* and exacerbate the zoonotic risk (Ahmed et al. 2019; da Silva et al. 2021). The spread of *B. coli* in confined pigs should then be controlled by monitoring their health status, providing hygienic drinking water and implementing biosecurity and hygiene measures throughout the fattening cycle.

## Conclusions

A high prevalence of *B. coli* was demonstrated in pigs raised in northern Italy and the sedimentation was found to be a more sensitive copromicroscopic method than the FLOTAC® technique. Sequence analysis revealed the presence of both ITS genetic types (A and B), which were simultaneously found in one sample. The most common sequence variant was B0, which was either unique or mixed with others (mainly B1).

These results provide current data on the circulation and molecular characterisation of *B. coli* in several pig farms during the fattening cycle, giving useful information for measures to contain the spread of this protist in pigs. Further studies are needed to determine its distribution in other pig production categories and in humans, associating any clinical symptomatology and individual susceptibility and correlating the actual role of pigs, since one of the main routes of transmission to humans is the direct contact with pig faeces. In-depth analyses should also be conducted to assess the survival of *B. coli* cysts in the environment and in food and water matrices, evaluating other sources of infections for both pigs and humans.

## Supplementary Information

Below is the link to the electronic supplementary material.

Supplementary file1 (PDF 114 KB)

Supplementary file2 (PDF 173 KB)

Supplementary file3 (PDF 182 KB)

Supplementary file4 (DOCX 20 KB)

## Data Availability

The datasets analysed during the current study are available from the corresponding authors on reasonable request.
