# Supplementary material for: Prevalence and molecular characterisation of Balantioides coli in pigs raised in Italy
Source: Parasitol Res. 2025 Jan 16;124(1):6. doi: 10.1007/s00436-025-08452-w (PMC11735580; doi:10.1007/s00436-025-08452-w)
Supplement: Supplementary file 1 — Supplementary file1 (PDF 114 KB) [file 436_2025_8452_MOESM1_ESM.pdf]

Suppl. File 1 Primers and cycling conditions used for molecular characterisation of *B. coli* in the present study**.**

Primers targeting the 3' end (~ 117 bp) of the SSU-rRNA gene, the ITS region and the start of the 5' end (~28 bp) of the LSU-rRNA gene of *B. coli*:

1. Forward primer B5D (5'-GCTCCTACCGATACCGGGT-3')
2. Reverse primer B5RC (5'-GCGGGTCATCTTACTTGATTTC-3')

Cycling conditions:

- Initial denaturation for 10 min at 94 ºC
- 30 cycles of denaturation for 1 min at 94 ºC, primer annealing for 1 min at 55 ºC, extension for 3 min at 72 ºC
- Final extension for 5 min at 72 °C
