# Supplementary material for: Prevalence and molecular characterisation of Balantioides coli in pigs raised in Italy
Source: Parasitol Res. 2025 Jan 16;124(1):6. doi: 10.1007/s00436-025-08452-w (PMC11735580; doi:10.1007/s00436-025-08452-w)

Suppl. File 2 Histogram showing the number of pig positive samples for *Balantioides coli* by sedimentation technique at the farm level in the two sampling sessions.

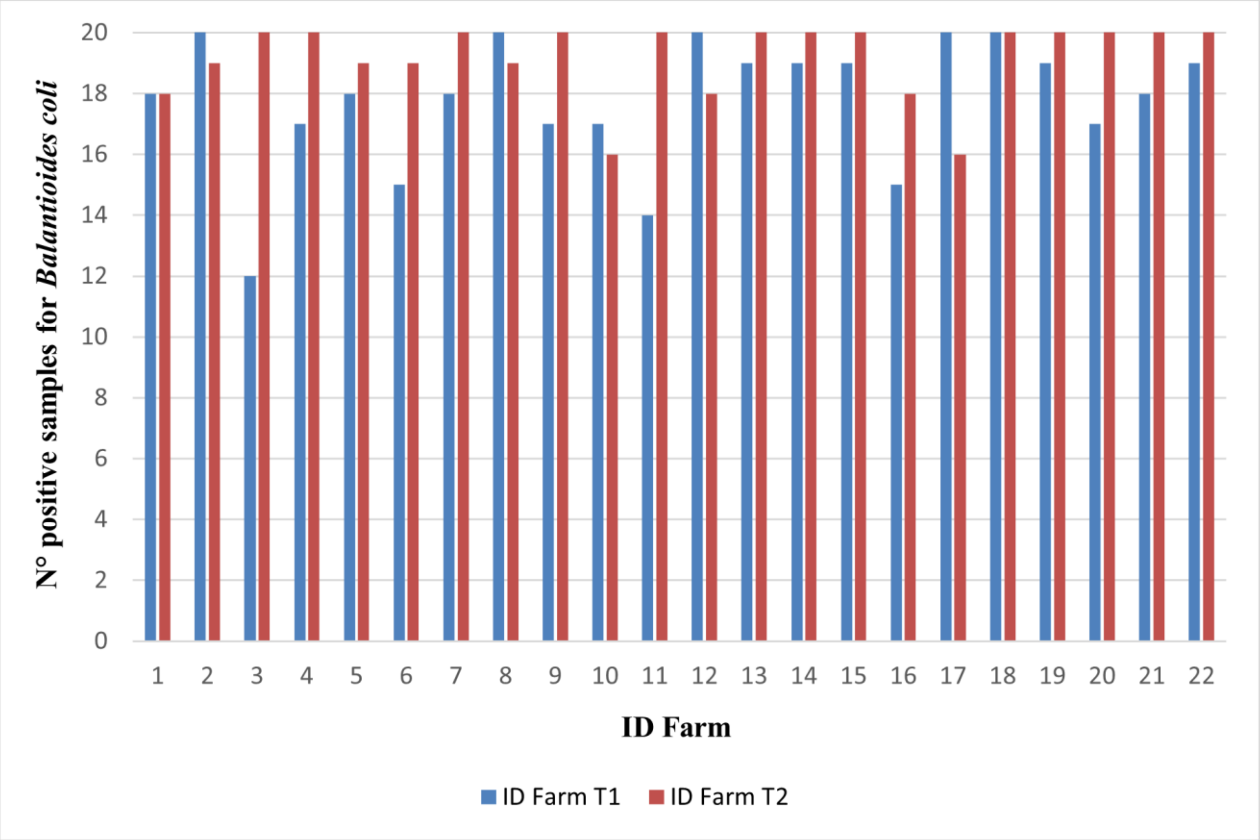

Supplement: Supplementary file 2 — Supplementary file2 (PDF 173 KB) [file 436_2025_8452_MOESM2_ESM.pdf]
