# Supplementary material for: Prevalence and molecular characterisation of Balantioides coli in pigs raised in Italy
Source: Parasitol Res. 2025 Jan 16;124(1):6. doi: 10.1007/s00436-025-08452-w (PMC11735580; doi:10.1007/s00436-025-08452-w)
Supplement: Supplementary file 3 — Supplementary file3 (PDF 182 KB) [file 436_2025_8452_MOESM3_ESM.pdf]

Suppl. File 3 Alignment of the ITS1 – 5.8s-rRNA – ITS2 sequences of *Balantioides coli* from Italian pigs obtained in this study. Sequences of *B. coli* JF444262-JF44465 and *Buxtonella sulcata* JQ073387 were included for comparative purposes. Sequence *B. coli* JF444762 was used as reference.

```
                                     10        20        30       40          50        60        70        80        90
                             -------- -+---------+---------+--------+---------  +---------+---------+---------+---------+--------
B. coli JF444762 (A0)        TTAACAAA-ATTTGAACTAATAACTAACTGAATTTTATAGTGAAATTTA--TTTCACTATATTTGAACTAATATAATAAAACCATAAACTACTTAATTGT   97
B. coli JF444763 (A1)        ........-.......................C..G........G....TT.................................................   99
B. coli JF444759 (A2)        ........-.......................C..G.............TT.................................................   99
B. coli JF444764 (B0)        ........-.C..............G......C..G.......GCAA------........C...........C.....C....C..T.C.......-..   92
B. coli JF444765 (B1)        ........-.A..............G......C..G.......GTAA------........C...........C.....C....C..T.C.......-..   92
L1 (PP888164)                ........-.C..............G......C..G.......GCAA------........C...........C.....C....C..T.C.......-..   92
P1 (PP888165)                ........-.C..............G......C..G.......GCAA------........C...........C.....C....C..T.C.......-..   92
P2 (PP888166)                ........-.C..............G......C..G.......GYAA------........C...........C.....C....C..T.C.......-..   92
L2 (PP888167)                ........-.M..............G......C..G.......GYAA------........C...........C.....C....C..T.C.......-..   92
L3 (PP888168)                ........-.M..............G......C..G.......GYAA------........C...........C.....C....C..T.C.......-..   92
P3 (PP888169)                ........-.M..............G......C..G.......GYAA------........C...........C.....C....C..T.C.......-..   92
L4 (PP888170)                ........-.C..............G......C..G.......GYAA------........C...........C.....C....C..T.C.......-..   92
L5 (PP888171)                ........-.M..............G......C..G.......GYAA------........C...........C.....C....C..T.C.......-..   92
L6 (PP888172)                ........-.M..............G......C..G.......GYAA------........C...........C.....C....C..T.C.......-..   92
L7 (PP888173)                ........-.M..............G......C..G.......GYAA------........C...........C.....C....C..T.C.......-..   92
L8 (PP888174)                ........-.M..............G......C..G.......GYAA------........C...........C.....C....C..T.C.......-..   92
L9 (PP888175)                ........-........................................--............................................W....   97
L10 (PP888176)               ........-.C..............G......C..G.......GCAA------........C...........C.....C....C..T.C.......-..   92
L11 (PP888177)               ........-.C..............G......C..G.......GCAA------........C...........C.....C....C..T.C.......-..   92
L12 (PP888178)               ........-.C..............G......C..G.......GCAA------........C...........C.....C....C..T.C.......-..   92
L13 (PP888179)               ........-.C..............G......C..G.......GCAA------........C...........C.....C....C..T.C.......-..   92
ER1 (PP888180)               ........-.C..............G......C..G.......GCAA------........C...........C.....C....C..T.C.......-..   92
L14 (PP888181)               ........-.M..............G......C..G.......GYAA------........C...........C.....C....C..T.C.......-..   92
L15 (PP888182)               ........-........................................--.................................................   97
L15 (PP888183)               ........-.A..............G......C..G.......GTAA------........C...........C.....C....C..T.C.......-..   92
L16 (PP888184)               ........-.C..............G......C..G.......GCAA------........C...........C.....C....C..T.C.......-..   92
L17 (PP888185)               ........-........................................--.................................................   97
L18 (PP888186)               ........-.M..............G......C..G.......GYAA------........C...........C.....C....C..T.Y.......-..   92
B. sulcata JQ073387          ..T....GC.A............T.......T.AC.CATAGT-.GG..CT--CC.TACTAT-..TG.ACT.ATC.T.T..T..CAT...CCA..T.A.T.   96
```

```
                      100       110       120       130       140       150       160       170       180       190
                      +---------+---------+---------+---------+---------+---------+---------+---------+---------+---------
B. coli JF444762 (A0) TTGAATATCAATCTAAATTTTCAACGATGGATGTCTTGGCTCCCATATCGATGAAGAACGCAGCAAGAATGCGATATGCAATGTGAATTGCAGAACCATG  196
B. coli JF444763 (A1) ....................................................................................................  198
B. coli JF444759 (A2) ....................................................................................................  198
B. coli JF444764 (B0) ....................................................................................................  191
B. coli JF444765 (B1) ....................................................................................................  191
L1 (PP888164)         ....................................................................................................  191
P1 (PP888165)         ....................................................................................................  191
P2 (PP888166)         ....................................................................................................  191
L2 (PP888167)         ....................................................................................................  191
L3 (PP888168)         ....................................................................................................  191
P3 (PP888169)         ....................................................................................................  191
L4 (PP888170)         ....................................................................................................  191
L5 (PP888171)         ....................................................................................................  191
L6 (PP888172)         ....................................................................................................  191
L7 (PP888173)         ....................................................................................................  191
L8 (PP888174)         ....................................................................................................  191
L9 (PP888175)         ....................................................................................................  196
L10 (PP888176)        ....................................................................................................  191
L11 (PP888177)        ....................................................................................................  191
L12 (PP888178)        ....................................................................................................  191
L13 (PP888179)        ....................................................................................................  191
ER1 (PP888180)        ....................................................................................................  191
L14 (PP888181)        ....................................................................................................  191
L15 (PP888182)        ....................................................................................................  196
L15 (PP888183)        ....................................................................................................  191
L16 (PP888184)        ....................................................................................................  191
L17 (PP888185)        ....................................................................................................  196
L18 (PP888186)        ....................................................................................................  191
B. sulcata JQ073387   .G.TG.ATAA-T........................................................................................  194
```

```
                      200       210       220       230        240       250       260       270       280       290
                      +---------+---------+---------+--- ------+---------+---------+---------+---------+---------+--------
B. coli JF444762 (A0) AATCATCGGATATTCTAACGCAACTGGCACTGGC-TAAACCAGTATACTTGTTTCAGTGTGCGTAACCAAACATATAATCAAAATGTAAGAGATCATCTC  296
B. coli JF444763 (A1) ..................................-...........................................A.....................  298
B. coli JF444759 (A2) ..................................-.........................................T.A.....................  298
B. coli JF444764 (B0) ..................................-......................................C.C....................C...  291
B. coli JF444765 (B1) ..................................-......................................C.C....................C...  291
L1 (PP888164)         ..................................-......................................C.C....................C...  291
P1 (PP888165)         ..................................-......................................C.C..N.................C...  291
P2 (PP888166)         ..................................-......................................C.C..W.................C...  291
L2 (PP888167)         ..................................-......................................C.C..W.................C...  291
L3 (PP888168)         ..................................-......................................C.C..W.................C...  291
P3 (PP888169)         ..................................-......................................C.C..W.................C...  291
L4 (PP888170)         ..................................-......................................Y.C..W.................C...  291
L5 (PP888171)         ..................................-......................................C.C..W.................C...  291
L6 (PP888172)         ..................................-......................................C.C..W.................C...  291
L7 (PP888173)         ..................................-......................................C.C..W.................C...  291
L8 (PP888174)         ..................................-......................................C.C..W.................C...  291
L9 (PP888175)         ..................................-...........................................W.....................  296
L10 (PP888176)        ..................................-......................................C.C..W.................C...  291
L11 (PP888177)        ..................................-......................................C.C..W.................C...  291
L12 (PP888178)        ..................................-......................................C.C..W.................C...  291
L13 (PP888179)        ..................................-......................................C.C..W.................C...  291
ER1 (PP888180)        ..................................-......................................C.C..W.................C...  291
L14 (PP888181)        ..................................-......................................C.C..W.................C...  291
L15 (PP888182)        ..................................-.................................................................  296
L15 (PP888183)        ..................................-......................................CGC....................C...  291
L16 (PP888184)        ..................................-......................................C.C..W.................C...  291
L17 (PP888185)        ..................................-...........................................W.....................  296
L18 (PP888186)        ..................................-......................................C.C..W.................C...  292
B. sulcata JQ073387   ...........T.....................AT.............................C.A..TT...T...CT.T..................  295
```

```
                           300       310       320       330       340       350       360
                          -+---------+---------+---------+---------+---------+---------+--------
B. coli JF444762 (A0)     TTATTAAATAAATGCTCATTGTAGCAATCACAATATTTTTGTGAATTTACATTGAGATAATTTTTAATTT  366
B. coli JF444763 (A1)     ......................................................................  368
B. coli JF444759 (A2)     ......................................................................  368
B. coli JF444764 (B0)     ....G....G........................-C...........-..TC.....C--....C.....  357
B. coli JF444765 (B1)     ....G....G........................-C...........-..TC.....C--....C.....  357
L1 (PP888164)             ....G....G........................-C...........-..TC.....C--....C.....  357
P1 (PP888165)             ....G....G........................-C...........-..TC.....C--....C.....  357
P2 (PP888166)             ....G....G........................-C...........-..TC.....C--....C.....  357
L2 (PP888167)             ....G....G........................-C...........-..TC.....C--....C.....  357
L3 (PP888168)             ....G....G........................-C...........-..TC.....C--....C.....  357
P3 (PP888169)             ....G....G........................-C...........-..TC.....C--....C.....  357
L4 (PP888170)             ....G....G........................-C...........-..TC.....C--....C.....  357
L5 (PP888171)             ....G....G........................-C...........-..TC.....C--....C.....  357
L6 (PP888172)             ....G....G........................-C...........-..TC.....C--....C.....  357
L7 (PP888173)             ....G....G........................-C...........-..TC.....C--....C.....  357
L8 (PP888174)             ....G....G........................-C...........-..TC.....C--....C.....  357
L9 (PP888175)             ................................................................-.....  365
L10 (PP888176)            ....G....G........................-C...........-..TC.....C--....C.....  357
L11 (PP888177)            ....G....G........................-C...........-..TC.....C--....C.....  357
L12 (PP888178)            ....G....G........................-C...........-..TC.....C--....C.....  357
L13 (PP888179)            ....G....G........................-C...........-..TC.....C--....C.....  357
ER1 (PP888180)            ....G....G........................-C...........-..TC.....C--....C.....  357
L14 (PP888181)            ....G....G........................-C...........-..TC.....C--....C.....  357
L15 (PP888182)            ................................................................-.....  365
L15 (PP888183)            ....G..C.G........................-C...........-..TC.....C--....C.....  357
L16 (PP888184)            ....G....G........................-C...........-..TC.....C--....C.....  357
L17 (PP888185)            ................................................................-.....  365
L18 (PP888186)            ....G....G........................-C...........-..TC.....C--....C.....  357
B. sulcata JQ073387       ....G....G.......T........G.....T.-.C........AA....AAA....T...A.-.C...  363
```
