# Supplementary material for: Prevalence and molecular characterisation of Balantioides coli in pigs raised in Italy
Source: Parasitol Res. 2025 Jan 16;124(1):6. doi: 10.1007/s00436-025-08452-w (PMC11735580; doi:10.1007/s00436-025-08452-w)
Supplement: Supplementary file 4 — Supplementary file4 (DOCX 20 KB) [file 436_2025_8452_MOESM4_ESM.docx]

**Suppl. Table 1** Farm management characteristics, animal cleanliness scores and distribution of *Balantioides coli* positive samples using the sedimentation technique in two different sampling sessions in pig farms in Italy.

| **Farm code** | **Farm management characteristics** | | | **Animal cleanliness score** | **T1**  **N° Positive samples/Total** | **T2**  **N° Positive samples/Total** |
| --- | --- | --- | --- | --- | --- | --- |
|  | All-in/All-out system | Type of floor | Type of watering |  | **Prevalence (95% CI^a^)** | **Prevalence (95% CI)** |
| 1 | Yes | Mixed | Well water | 1 | 18/20 | 18/20 |
|  |  |  |  |  | 90% (68.3-98.8) | 90% (68.3-98.8) |
| 2 | Yes | Full | Acqueduct | 1 | 20/20 | 19/20 |
|  |  |  |  |  | 100% (83.2-100) | 95% (75.1-99.9) |
| 3 | No | Full | Well water | 2 | 12/20 | 20/20 |
|  |  |  |  |  | 60% (36-80.9) | 100% (83.2-100) |
| 4 | No | Full | Well water | 2 | 17/20 | 20/20 |
|  |  |  |  |  | 85% (62.1-96.8) | 100% (83.2-100) |
| 5 | Yes | Slatted | Well water | 1 | 18/20 | 19/20 |
|  |  |  |  |  | 90% (68.3-98.8) | 95% (75.1-99.9) |
| 6 | No | Full | Well water | 2 | 15/20 | 19/20 |
|  |  |  |  |  | 75% (50.9-91.3) | 95% (75.1-99.9) |
| 7 | Yes | Full | Well water | 1 | 18/20 | 20/20 |
|  |  |  |  |  | 90% (68.3-98.8) | 100% (83.2-100) |
| 8 | Yes | Slatted | Well water | 0 | 20/20 | 19/20 |
|  |  |  |  |  | 100% (83.2-100) | 95% (75.1-99.9) |
| 9 | No | Full | Well water | 2 | 17/20 | 20/20 |
|  |  |  |  |  | 85% (62.1-96.8) | 100% (83.2-100) |
| 10 | Yes | Slatted | Acqueduct | 0 | 17/20 | 16/20 |
|  |  |  |  |  | 85% (62.1-96.8) | 80% (56.3-94.3) |
| 11 | No | Full | Well water | 2 | 14/20 | 20/20 |
|  |  |  |  |  | 70% (45.7-88.1) | 100% (83.2-100) |
| 12 | Yes | Slatted | Well water | 0 | 20/20 | 18/20 |
|  |  |  |  |  | 100% (83.2-100) | 90% (68.3-98.8) |
| 13 | Yes | Mixed | Well water | 1 | 19/20 | 20/20 |
|  |  |  |  |  | 95% (75.1-99.9) | 100% (83.2-100) |
| 14 | Yes | Slatted | Well water | 1 | 19/20 | 20/20 |
|  |  |  |  |  | 95% (75.1-99.9) | 100% (83.2-100) |
| 15 | Yes | Slatted | Well water | 1 | 19/20 | 20/20 |
|  |  |  |  |  | 95% (75.1-99.9) | 100% (83.2-100) |
| 16 | No | Full | Well water | 1 | 15/20 | 18/20 |
|  |  |  |  |  | 75% (50.9-91.3) | 90% (68.3-98.8) |
| 17 | Yes | Slatted | Well water | 1 | 20/20 | 16/20 |
|  |  |  |  |  | 100% (83.2-100) | 80% (56.3-94.3) |
| 18 | Yes | Slatted | Well water | 1 | 20/20 | 20/20 |
|  |  |  |  |  | 100% (83.2-100) | 100% (83.2-100) |
| 19 | Yes | Full | Well water | 1 | 19/20 | 20/20 |
|  |  |  |  |  | 95% (75.1-99.9) | 100% (83.2-100) |
| 20 | Yes | Full | Well water | 1 | 17/20 | 20/20 |
|  |  |  |  |  | 85% (62.1-96.8) | 100% (83.2-100) |
| 21 | Yes | Full | Well water | 1 | 18/20 | 20/20 |
|  |  |  |  |  | 90% (68.3-98.8) | 100% (83.2-100) |
| 22 | Yes | Full | Well water | 1 | 19/20 | 20/20 |
|  |  |  |  |  | 95% (75.1-99.9) | 100% (83.2-100) |
| **Total** | | | | | **391/440** | **422/440** |
|  |  |  |  |  | **88.9% (85.5-91.6)** | **95.9% (93.6-97.6)** |

^a^CI: Confidence interval
